# A Case of Epleno-Medullary Leukemia Treated with Benzol

**Published:** 1914-06

**Authors:** 


**A Case of Epleno-Medullary Leukemia Treated with Benzol.** Dr. Victor Fossati, La Semana Medica, September 11, 1913, reports the case of a girl, 22 years old, who for a year had noticed a swelling in her abdominal cavity which increased rapidly in size. She had periodical attacks of fever, intense pain in her left side, lost flesh and strength and became very anaemic. On examination, the spleen was found to be enormously enlarged, but there was no general hypertrophy.

Examination of the blood:

| | |
|---|---|
| Red cells | 2,000,000 |
| Whites | 240,000 |
| Haemoglobin | 37 |
| Polys | 45 |
| Eosinophiles | 3 |
| Lymphocytes | 35 |
| Large mononuclears | 8 |
| Myelocytes | 9 |

The treatment was started with iron and arsenic and X-Rays. No benefit accrued. On March 9th benzol in creatin coated capsules was given—20 drops a day—increased day by day to 70 drops. The general condition of the patient steadily improved. However the patient suffered greatly from gastro-intestinal symptoms, due to the benzol, and its administration had to be stopped from time to time. The spleen showed no marked reduction in size.

Blood examination, October 6, 1913:

| | |
|---|---|
| Red cells | 3,000,000 |
| Whites | 56,000 |
| Polys | 59 |
| Eosinophiles | 1 |
| Lymphocytes | 30 |
| Large mononuclears | 3 |
| Myelocytes | 6 |

The pains have ceased, the appetite is good; the patient attends to her ordinary duties and continues to take the benzol.

